# Specificity of Morbillivirus Hemagglutinins to Recognize SLAM of Different Species

**DOI:** 10.3390/v11080761

**Published:** 2019-08-19

**Authors:** Hideo Fukuhara, Yuri Ito, Miyuki Sako, Mizuho Kajikawa, Koki Yoshida, Fumio Seki, Mwila Hilton Mwaba, Takao Hashiguchi, Masa-aki Higashibata, Toyoyuki Ose, Kimiko Kuroki, Makoto Takeda, Katsumi Maenaka

**Affiliations:** 1 Center for Research and Education on Drug Discovery, Hokkaido University, Kita-12, Nishi-6, Kita-ku, Sapporo 060-0812, Japan; 2 Laboratory of Biomolecular Science, Faculty of Pharmaceutical Sciences, Hokkaido University, Kita-12, Nishi-6, Kita-ku, Sapporo 060-0812, Japan; 3 Medical Institute of Bioregulation, Kyushu University, Fukuoka 812-8582, Japan; 4 Department of Virology 3, National Institute of Infectious Diseases, Tokyo 208-0011, Japan; 5 Department of Virology, Faculty of Medicine, Kyushu University, Fukuoka 812-8582, Japan; 6 Core Research for Evolutional Science and Technology (CREST), Japan Science and Technology Agency, 4-1-8, Honcho Kawaguchi, Saitama 332-0012, Japan

**Keywords:** morbillivirus, hemagglutinin, SLAM, canine distemper virus, measles virus, surface plasmon resonance, structure

## Abstract

Measles virus (MV) and canine distemper virus (CDV) are highly contagious and deadly, forming part of the morbillivirus genus. The receptor recognition by morbillivirus hemagglutinin (H) is important for determining tissue tropism and host range. Recent reports largely urge caution as regards to the potential expansion of host specificities of morbilliviruses. Nonetheless, the receptor-binding potential in different species of morbillivirus H proteins is largely unknown. Herein, we show that the CDV-H protein binds to the dog signaling lymphocyte activation molecule (SLAM), but not to the human, tamarin, or mouse SLAM. In contrast, MV-H can bind to human, tamarin and dog SLAM, but not to that of mice. Notably, MV binding to dog SLAM showed a lower affinity and faster kinetics than that of human SLAM, and MV exhibits a similar entry activity in dog SLAM- and human SLAM-expressing Vero cells. The mutagenesis study using a fusion assay, based on the MV-H–SLAM complex structure, revealed differences in tolerance for the receptor specificity between MV-H and CDV-H. These results provide insights into H-SLAM specificity related to potential host expansion.

## 1. Introduction

Morbilliviruses are extremely contagious viruses, belonging to the non-segmented, negative-stranded RNA group of viruses. Morbilliviruses include the measles virus (MV), canine distemper virus (CDV), and rinderpest virus (RPV). CDV had been assumed to infect only carnivores, including dogs. However, recent studies have reported the expansion of host specificities beyond the previously conceived natural hosts [1,2,3]. Therefore, the understanding and prediction of host specificity of morbilliviruses are becoming increasingly important for preventing outbreaks, as the current vaccines for MV, CDV, and RPV are highly effective.

The entry step to the host cells is very important for determining the host range and tissue tropisms for these viruses [4]. Morbilliviruses have two types of glycoproteins, namely hemagglutinins (H) and fusion (F) proteins on the viral surface. In the first step of invasion, an H protein binds to the entry receptor, the signaling lymphocyte activation molecule (SLAM, also known as CD150) is expressed on immune cells, and induces conformational change in the accompanying F protein, which fuses the viral membrane with the plasma membrane of the target immune cells [5,6,7]. After adequate multiplication of viruses, coupled with the control of host immune responses in the whole body, MV-infected immune cells fuse with epithelial cells using nectin 4 (PVRL4) on the basal side of epithelial cells, and the transfer of the viral components to the epithelial cells, from which progeny virus particles are released efficiently to outside cells [8,9,10,11]. All morbilliviruses exhibit host specificity [4,12]. H and SLAM interactions are responsible for determining the host specificity during the entry events. Although, many infection and fusion studies have reported SLAM-mediated host specificity, as well as some binding studies for SLAM-H interactions [13,14,15], there is limited information regarding other host species. In this study, we revealed the molecular basis for host specificity of the morbillivirus, through the interspecific study for SLAM binding to H proteins, using recombinant SLAM proteins from a set of species (humans, tamarins, dogs, and mice), as well as recombinant H proteins from MV and CDV. We performed a comprehensive binding study for SLAM-H interactions, using surface plasmon resonance (SPR), and examined the recognition interface through a mutagenesis study.

## 2. Materials and Methods

### 2.1. Preparation of Recombinant Proteins

The head domain of MV-H from IC-B wild-type and Edmonston vaccine strains were prepared using the HEK 293T cell (ATCC, www.atcc.org) expression system, as previously described [14]. The head domain of CDV hemagglutinin (CDV-H) from wild-type and vaccine strains were also prepared in the same way. Briefly, the cDNAs, encoding the head domain (Asn149–Arg604) of CDV-H from the 5VD strain, were amplified using 5′-TGCGTAGCTAGCAATTTTACTAATTACTGTGATGC-3′ as the forward primer and 5′-CAGAACCTCGAGACGATTACATGAGAATCTTATACGG-3′ as the reverse primer, respectively. The primer sets of 5′- TGCGTAGCTAGCAATTTTACTAATTACTGTGAGTC-3′ and 5′-CAGAACCTCGAGACGGTTACATGAGAATCTCATACGG-3′ were used to amplify the DNA coding the head domain (Asn149–Arg604) of CDV-H from the vaccine (Kyoto Biken Laboratories, Inc., Kyoto, Japan) strain. The amplified DNA fragments were sub-cloned into *Nhe* I-*Xho* I restriction sites on a derivative of the mammalian expression vector pCA7 (a modified pCAGGS vector) to fuse a secretion signal of tyrosine phosphatase and biotinylation tag at the N-terminus, and hexa-histidine tag at the C-terminus [16]. The expression plasmids were transfected by polyethyleneimine max (Polysciences, Inc., Warrington, PA, USA) into HEK 293T cells. After transfection, the cells were cultured in D-MEM (Dulbecco’s Modified Eagle Medium) (FUJIFILM Wako Pure Chemical Corporation, Osaka, Japan) supplemented with 2% FCS (Fetal Calf Serum) (GE Healthcare UK Ltd, Buckinghamshire, England) at 5% CO_2_ for 4 days. The soluble proteins, secreted into the culture medium, were purified using Ni^2+^-affinity resin, HisTrap (GE Healthcare UK Ltd, Buckinghamshire, England), and gel filtration with a superdex 200 GL 10/300 column (GE Healthcare UK Ltd, Buckinghamshire, England) in the gel filtration buffer (20 mM Tris-HCl pH 8.0, 150 mM NaCl), following the biotinylation using BirA biotin ligase (Avidity LLC, Aurora, CO, USA). The peak fractions, containing soluble H protein, were pooled and the purity was confirmed by sodium dodecyl sulfate polyacrylamide gel electrophoresis (SDS-PAGE).

The soluble human SLAM (hSLAM) was expressed as a chimeric protein of a human V-set with a mouse C2 domain as previously described [14]. The soluble SLAM ectodomains (V-set and C2) from a cotton-top tamarin and mouse were prepared, as previously described [14,17]. Soluble dog SLAM (dSLAM) was also prepared in a similar way to the other SLAM proteins. Briefly, the cDNA encoding the extracellular domain (Met1-Leu240) of dSLAM, including its authentic signal sequence, was amplified using primer sets of 5′- GGCAAAGAATTCGCCACCATGGATTCCAGGGGCTTC-3′ with 5′-ATGCAGGGTACCCAGTCTCCATTGTCTTGGCACCG-3′ and sub-cloned into a pCA7 vector, with the *Eco* RI-*Kpn* I site to fuse a C-terminal hexa-histidine tag. HEK 293T cells were transfected with the plasmid DNA and maintained for a further 4 days. The culture supernatant was collected and subsequently purified, using HisTrap and Superdex200 10/300GL column (GE Healthcare UK Ltd, Buckinghamshire, England).

### 2.2. Binding Analysis

To investigate the specificity and cross-reactivity of MV-H and CDV-H against different species of SLAM proteins, SPR experiments were performed using the BIAcore 3000 system (GE Healthcare UK Ltd, Buckinghamshire, England) at 25 °C. To avoid the avidity effects of H protein dimers, biotinylated H proteins (500–1600 RU) were immobilized on a CAP chip (GE Healthcare UK Ltd, Buckinghamshire, England) as ligands. Biotinylated human leukocyte antigen (HLA)-G (2300 RU) and BSA (380 RU) were also immobilized as negative controls. For specificity analysis, each monomeric SLAM protein was diluted with HBS-EP buffer (0.01 M HEPES pH 7.4, 0.15 M NaCl, 0.005% (*v*/*v*) Surfactant P20) to 10 μM and injected over immobilized ligands at a flow rate of 5 µL/min. The binding response was calculated by subtracting the response of the negative control from that of the H protein flow cell. A kinetic analysis was performed at lower immobilization levels (50–350 RU). The binding response at each concentration of SLAM (0.25, 0.5, and 1.0 µM) was calculated by subtracting the equilibrium response, measured in the control flow cell, from the response in each sample flow cell. Only for dog SLAM, a series of 0.3, 0.6, and 1.2 µM were used toward wild-type MV-H. The kinetic parameters were calculated using the curve-fitting program of the BIAevaluation version 4.2 software (GE Healthcare UK Ltd, Buckinghamshire, England) to fit the rate equations derived from the simple 1:1 Langmuir binding model (A + B ↔ AB).

### 2.3. Cell-to-Cell Fusion Assay

To confirm the capability of different SLAM receptor usage by MV- and CDV-H, we performed a cell-to-cell fusion analysis. DNA fragments, encoding the full-length of the H and F protein of the wild-type MV and CDV, were each sub-cloned into a pCA7 vector [16]. Vero/hSLAM and Vero.DogSLAMtag cells, in a 48-well plate, were co-transfected by 0.6 µg of polyethyleneimine max with the combination of 0.1 µg of H, F, and mCherry-expressing plasmids [18,19]. Since the vaccine strains of MV and CDV can fuse parental Vero cells, we used the combination of H and F proteins of the IC-B strain for wild-type MV, and the A75/17 strain for CDV. Fluorescent images were acquired with a BZ-X700 fluorescent microscope (Keyence Corporation, Osaka, Japan) with a 2× objective at 42 h post-transfection time.

### 2.4. Infectious Assay Using Recombinant Measles Virus and Canine Distemper Virus

Vero/hSLAM, Vero.DogSLAMtag, and parental Vero cells in a 24-well plate were infected with the MV IC323 strain (recombinant wild-type) or CDV Ac96I (wild isolate) at a multiplicity of infection of 0.01 per cell [20,21]. The culture supernatants were obtained at various time intervals. The MV titers were determined with Vero/hSLAM cells by plaque forming assay, and the CDV titers were determined with Vero.DogSLAMtag cells by 50% tissue culture infectious dose (TCID_50_) assay [22].

## 3. Results and Discussion

**Figure 1:**
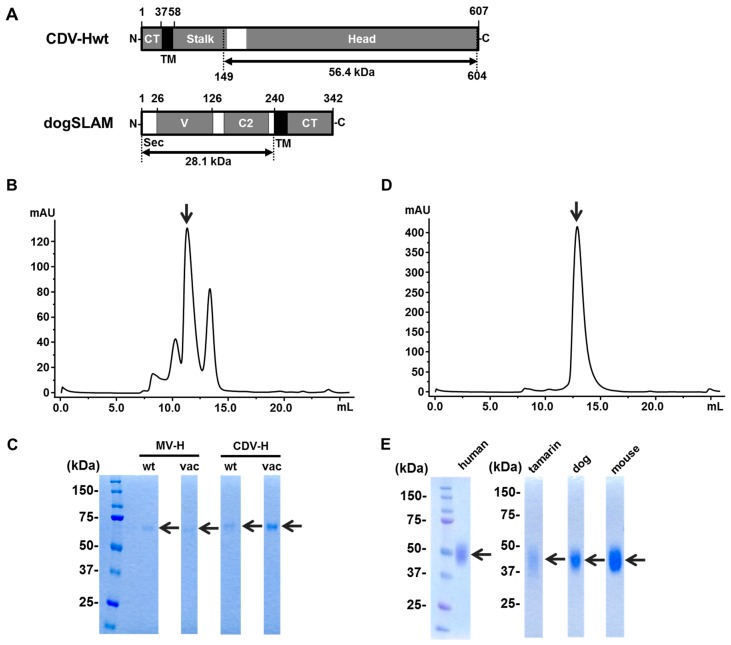
Expression and purification of canine distemper virus hemagglutinin (CDV-H) and dog signaling lymphocyte activation molecule (SLAM) proteins. (**A**) Schematic images for the domains of CDV-H (top) and dog SLAM (bottom). CT: Cytoplasmic tail, TM: Transmembrane domain, Head: Globular head domain, Sec: Secretary signal sequence, V: V-set domain. The arrows indicate the expression regions. (**B**) Gel filtration chromatograph of CDV-Hvac head domain. The arrow indicates the fraction of CDV-Hvac. (**C**) Sodium dodecyl sulfate polyacrylamide gel electrophoresis (SDS-PAGE) analysis of purified hemagglutinins (H) proteins (MV-Hwt, MV-Hvac, CDV-Hwt, and CDV-Hvac) in reduced condition. (**D**) Gel filtration chromatograph of dog SLAM. The arrow indicates the fraction containing soluble dog SLAM. (**E**) SDS-PAGE analysis of purified SLAM proteins (human, tamarin, dog, and mouse) in reduced condition. The arrows indicate the protein bands corresponding to each protein.

The CDV-H is a type II membrane protein and consists of an N-terminal cytoplasmic tail, a transmembrane region, a stalk region, and a C-terminal head domain (Figure 1A top). The study focused on two types of CDV-H proteins, derived from 5VD and Kyoto-Biken strains, representing the wild-type, and vaccine strains, respectively, hereafter designated CDV-Hwt and CDV-Hvac. We successfully produced the soluble head domains of the CDV-Hs with His-tag by transient expression using HEK 293T cells. The CDV-H proteins were purified by Ni-NTA (nickel-nitrilotriacetic acid) column chromatography following biotinylation and gel filtration. The recombinant proteins of the head domains (residues 149–617) of MV-Hwt (IC-B strain) and MV-Hvac (Edmonston strain) were prepared as biotinylated ligands using a similar method to that reported in [14].

The head domains (residues 149–604, 56.4kDa) of CDV-Hwt (5VD strain) and CDV-Hvac (Kyoto strain) were prepared in a similar way to MV-H. While the expected monomer size of the H proteins was approximately 60 kDa, the profile of size exclusion chromatography and SDS-PAGE showed that the H proteins form a ~120 kDa homodimer by disulfide bond (Figure 1B and 1C). The extracellular domain of dog SLAM (residues 1–240, 28.1kDa), fused with C-terminal His-tag, was transiently expressed by HEK 293T cell and purified in the same way as CDV-H proteins described above (Figure 1A bottom, 1D and 1E). The other soluble SLAM proteins (human, tamarin, and mouse) were produced as described previously [14,17].

**Figure 2:**
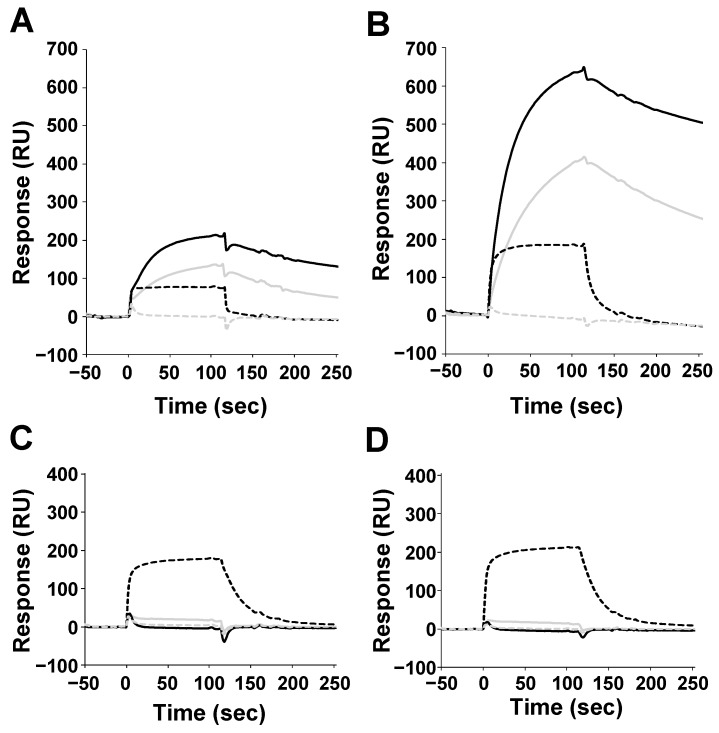
Binding of SLAM proteins to measles virus (MV) and CDV-H from the wild-type and vaccine strain. Human (gray solid line), tamarin (solid line), dog (broken line), and mouse SLAM (gray broken line) were injected for 120 s through flow cells bound with (**A**) MV-Hwt (641 RU), (**B**) MV-Hvac (1651 RU), (**C**) CDV-Hwt (574 RU), and (**D**) CDV-Hvac (776 RU). As a negative control, the response of HLA-G immobilized flow cells was subtracted.

**Figure 3:**
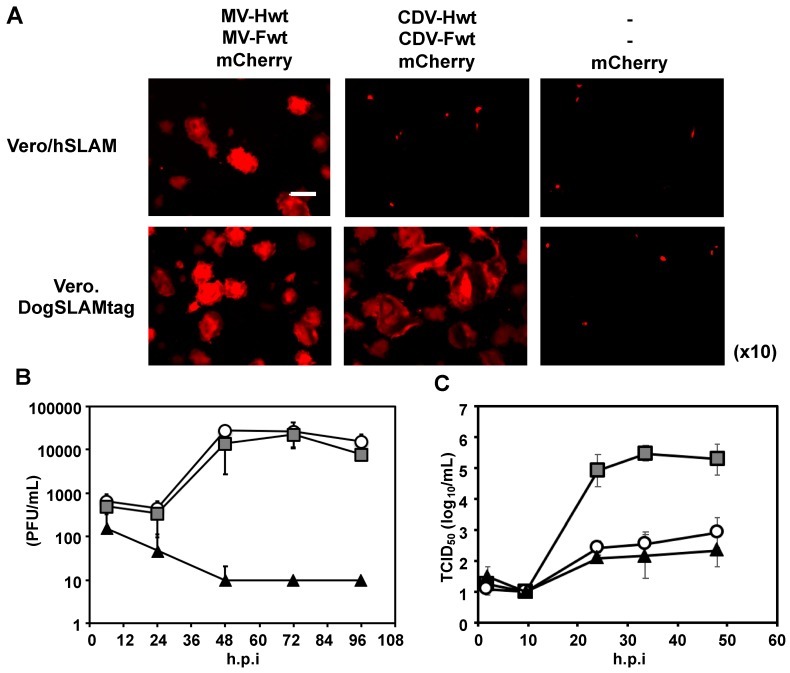
Infectious activity of MV toward Vero cells expressing human or dog SLAM. (**A**) Syncytium formation of SLAM-expressing Vero cells co-transfected with a combination of H, fusion (F), and mCherry expression plasmids. MV-H and -F of the wild-type IC-B strain, and CDV-H and -F of the wild-type A75/17 strain were used. Fluorescent images were acquired at 42 h after transfection. The scale bar indicates 400 µm. (**B**) Growth kinetics of the recombinant MV IC323 strain in Vero/hSLAM (open circles), Vero.DogSLAMtag (grey squares), and Vero cells (closed triangles). The data represent the means ± standard deviations of the results from triplicate samples. (**C**) The growth kinetics of the CDV Ac96I strain is shown in the same manner as Figure 3B. The data represent the means ± standard deviations of the results from triplicate samples.

Surface plasmon resonance (SPR analysis was performed using the above-mentioned H proteins (immobilized ligands) and SLAM proteins (analytes). Since the H proteins form homodimers, they were immobilized on the chip to avoid the avidity effect. Thus, these interactions can be treated as 1:1 binding. Consistent with previous reports, MV-H proteins showed binding responses to human and tamarin SLAM, but not to mouse SLAM (Figure 2A, B) [14]. Interestingly, MV-H also showed binding activity to the dog SLAM. To examine whether MV has dog-SLAM-mediated infectious activity, a fusion assay of MV toward the Vero cells expressing human or dog SLAM was performed (Figure 3A). Figure 3B shows that the wild-type MV (IC323 strain) has the ability to infect the Vero cells, that express dog SLAM at a level similar to that of the Vero cells expressing human SLAM; while the wide-type CDV (Ac96I strain) can infect dog SLAM-expressing Vero cells, but not human SLAM-expressing ones. This result indicates that MV has the potential to utilize dog SLAM to enter the cells, which might reasonably explain the report by Ryazantseva showing mild illness in puppies when inoculated by blood or throat washing from patients with measles [23]. On the other hand, CDV-H proteins could only bind to dog SLAM, and not to the human, tamarin, or mouse SLAM (Figure 2C, D and Figure 3A).

Next, we analyzed the kinetic parameters of H-SLAM interactions with a serial dilution of analytes, at low immobilization levels (50–350 RU) (Table 1, Figure S1). The dissociation constants (*K*_d_) of MV-H against human and tamarin SLAM were similar, and within the order of 10^−7^ ~ 10^−8^ M, whereas that of dog SLAM was in the order 10^−6^ ~ 10^−7^ M. This difference is caused by the *k*_on_ and *k*_off_ rates for dog SLAM, which were 10 times faster than that of the others (human and tamarin SLAM). On the other hand, the *K*_d_ value for CDV-H binding to dog SLAM (50–350 RU) was comparable to that of MV-H binding to human or tamarin SLAM, although the kinetics were both distinct and faster, when compared with the MV-H and human or tamarin SLAM interactions. The previous SPR study for CDV-H (A75/17 strain) binding to dog SLAM demonstrated similar results, with a somewhat lower affinity and faster dissociation [15] (Table 1).

**Figure 4:**
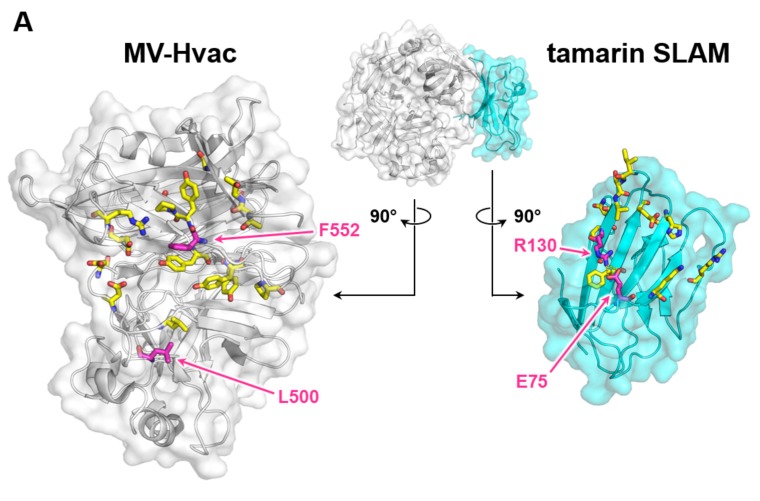

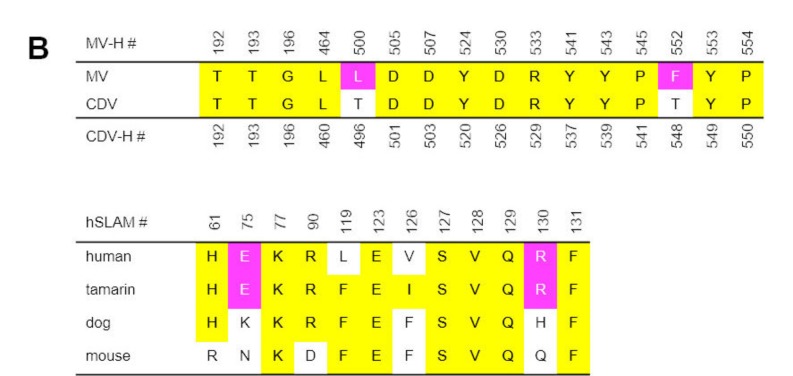
Conservation of H protein and SLAM interacting amino acid residues. (**A**) Mapping of conserved amino acids at the binding interface onto the crystal structure of the MV-Hvac monomer (light gray) in complex with tamarin SLAM (cyan) (Protein Data Bank (PDB) ID: 3ALW). Interacting amino acid residues are shown as yellow (conserved) or magenta (non-conserved) stick models. (**B**) Amino acid sequence alignments of the interacting residues, colored in the same way as Figure 4A. (**C**) Fusion activities for MV- and CDV-H mutants toward hSLAM and dSLAM. All figures are in the same scale and the white bar indicates 200 µm. The fused cell areas were measured using hybrid cell count software. The averaged values of MV-Hwt for Vero/hSLAM and CDV-Hwt for Vero.DogSLAMtag were set at 100%.

In the present study, MV-H was able to bind to human, tamarin, and dog SLAM, suggesting that MV-H recognizes the amino acid residues conserved among the three types of SLAM. Figure 4 shows the putative H-SLAM interface with amino acids, conserved among the MV-H and CDV-H proteins, (left) and those conserved among the SLAM species (human, tamarin, and dog, right). The structure and mapping were based on the crystal structure of the MV-H, complexed with tamarin SLAM (PDB ID: 3ALW) [17]. The mouse SLAM sequence is unique and differs considerably from the order of amino acids found in the other three species, explaining its inability to bind to MV-H and CDV-H. Dog SLAM exhibits some amino acid differences from human and tamarin ones, which likely cause faster kinetics and lower affinity than those found in the others. Previous studies have shown the importance of several residues, including H61 and E123 (conserved in the human, tamarin, dog, and mouse) for CDV-H binding and dog SLAM-dependent syncytia formation [15,17]. Additionally, other studies found amino acid residues 58 to 67 to be crucial in explaining MV-H–SLAM interactions in mice. R61H mutation in mouse SLAM was found to be vital while V60I and L63V mutations further increased mouse SLAM receptor–MV-H protein binding activity [24]. The presence of L63 in dog SLAM might explain the faster *k*_off_ rate compared to human and tamarin SLAM kinetics, as shown in Figure 2.

The majority of the MV-H recognition sites on SLAM (Figure 4A, yellow stick model) are highly conserved among human, tamarin, and dog species. This reasonably reflects the broad specificity of MV-H, as revealed by the SPR analysis. In contrast, the restricted specificity of CDV-H was thought to be dependent on the residues, T496 and T548 (magenta stick model in Figure 4A and outline characters in Figure 4B), which are different from the corresponding residues (L500 and F552) on MV-H. To investigate the contribution of these residues to H-SLAM interactions, we performed a fusion assay, using Vero cells expressing the CDV-type mutants for MV-H (L500T and F552T) or the MV-type mutants for CDV-H (T496L and T548F).

The F552T CDV-type mutants of MV-H showed a reduced fusion activity to human SLAM, and a slightly further reduction in activity to dog SLAM (Figure 4C), as well as L500T substitution. A previous study also found similar results, whereby F552 mutation marginally contributed to reduced fusion efficiency. While, another study showed the effect of F552V mutation in SLAM binding [14,25]. The partial effect of this mutation may have been due to its proximity to Y553, which was found to be among the anchor residues for SLAM binding [25]. However, this residue is conserved in CDV-H and MV-H (Figure 4B). These results indicate that the F552 of MV-H, to some extent, has the ability to confer fusion activities to both human and dog SLAM, thus raising the possibility that MV-H may have other human and dog SLAM binding sites, which determine the specificity, in addition to those observed in the complex structure.

The MV-type mutants of CDV-H (T496L and T548F) did not acquire the fusion ability to human SLAM, but unexpectedly, maintained the activity to dog SLAM (Figure 4C), while a reduction in the T548F mutation was observed. This is in line with previous studies, which found reduced SLAM-dependent fusion activity between mutant T548 and SLAM-expressing CHO (Chinese Hamster Ovary) cells [26]. However, studies by Zipperle et al. found that T548A mutation in the A75/17 wild-type strain only slightly affected SLAM binding and had little effect on fusion activity [27]. A further sequence comparison shows that some CDV isolates have a T548M substitution, though the impact of this substitution has not been investigated [28]. These results indicate that the amino acids T496 and T548 of CDV-H do not play a major role in the SLAM specificity or binding activity, suggesting that the dog SLAM binding mode of CDV-H is somehow different from that of the MV-H to hSLAM one. In addition, MV-Hvac shows higher affinities against human, tamarin, and dog SLAM than those of MV-Hwt (Table 1). No amino acid differences exist on the putative SLAM binding sites between MV-Hwt and MV-Hvac (Figure 4B), and thus the additional or indirect effects outside of the sites likely contribute to this difference.

With regards to the host range and virus spread between species, recent papers reported that, while CDV was previously believed to exhibit limited tropism for Carnivora, some strains of CDV can infect rhesus and cynomolgus monkeys [29,30,31]. Furthermore, CDV-H can adapt to human SLAM by only one mutation at position 540 [32]. Herein, we performed a binding study of CDV-H to the SLAM of different species, and tested two mutations on CDV-H based on the MV-H–SLAM complex structure, suggesting relatively strict tropism of CDV to dog SLAM. Some mutation sites allowing CDV-H to adapt to human SLAM, are located close to, but distinct from, the putative receptor-binding site. Therefore, these results suggest that CDV-H has an overlapping, but distinct SLAM-binding mode from MV-H, establishing a different receptor specificity from MV-H. These can acquire hSLAM and dSLAM fusion activities through additional effects. Further, structural studies for the H-SLAM complexes will reveal the detailed mechanisms of these recognitions, thus explaining host specificities.

## Figures and Tables

**Table 1:** Kinetic constants for the binding of H and SLAM proteins.

SLAM	Human			Tamarin			Dog			Mouse
	*K*_d_ [M]	*k*_on_ [M^−1^s^−1^]	*k*_off_ [s^−1^]	*K*_d_	*k*_on_	*k*_off_	*K*_d_	*k*_on_	*k*_off_	
MV-Hwt	4.48 × 10^−7^	1.41 × 10^4^	6.37 × 10^−3^	2.43 × 10^−7^	9.89 × 10^3^	2.41 × 10^−3^	1.35 × 10^−6^	7.21 × 10^4^	0.97 × 10^−1^	NB
MV-Hvac	6.08 × 10^−8^	4.79 × 10^4^	2.91 × 10^−3^	4.67 × 10^−8^	2.04 × 10^4^	9.52 × 10^−4^	7.16 × 10^−7^	1.33 × 10^5^	9.55 × 10^−2^	NB
CDV-Hwt	NB			NB			3.47 × 10^−7^	6.13 × 10^4^	2.40 × 10^−3^	NB
CDV-Hvac	NB			NB			2.44 × 10^−7^	1.13 × 10^5^	2.79 × 10^−2^	NB
CDV-A75/17-H [15]	NB			NB			8.0 × 10^−6^	2.5 × 10^4^	2.0 × 10^−1^	NB

NB: No binding.

## Supplementary Materials

The following are available online at https://www.mdpi.com/1999-4915/11/8/761/s1, Figure S1: Kinetic Analysis of SLAM proteins against MV-H and CDV-H from the wild-type and vaccine strain.

## References

[1] Terio K.A., Craft M.E. (2013). 2013 Canine Distemper Virus (CDV) in Another Big Cat: Should CDV Be Renamed Carnivore Distemper Virus?. MBio..

[2] Roelke-Parker M.E., Munson L., Packer C., Kock R., Cleaveland S., Carpenter M., O’Brien S.J., Pospischil A., Hofmann-Lehmann R., Lutz H. (1996). A canine distemper virus epidemic in Serengeti lions (Panthera leo). Nature.

[3] Nagao Y., Nishio Y., Shiomoda H., Tamaru S., Shimojima M., Goto M., Une Y., Sato A., Ikebe Y., Maeda K. (2011). An Outbreak of Canine Distemper Virus in Tigers (Panthera tigris): Possible Transmission from Wild Animals to Zoo Animals. J. Vet. Med. Sci..

[4] Yanagi Y., Takeda M., Ohno S. (2006). Measles virus: Cellular receptors, tropism and pathogenesis. J. Gen. Virol..

[5] Cocks B.G., Chang C.C., Carballido J.M., Yssel H., de Vries J.E., Aversa G. (1995). A novel receptor involved in T-cell activation. Nature.

[6] Tatsuo H., Ono N., Tanaka K., Yanagi Y. (2000). SLAM (CDw150) is a cellular receptor for measles virus. Nature.

[7] Tatsuo H., Ono N., Yanagi Y. (2001). Morbilliviruses use signaling lymphocyte activation molecules (CD150) as cellular receptors. J. Virol..

[8] Mühlebach M.D., Mateo M., Sinn P.L., Prüfer S., Uhlig K.M., Leonard V.H., Navaratnarajah C.K., Frenzke M., Wong X.X., Sawatsky B. (2011). Adherens junction protein nectin-4 is the epithelial receptor for measles virus. Nature.

[9] Noyce R.S., Bondre D.G., Ha M.N., Lin L.T., Sisson G., Tsao M.S., Richardson C.D. (2011). Tumor cell marker PVRL4 (Nectin-4) is an epithelial cell receptor for measles virus. PLoS Pathogens.

[10] Pratakpiriya W., Seki F., Otsuki N., Sakai K., Fukuhara H., Katamoto H., Hirai T., Maenaka K., Techangamsuwan S., Lan N.T. (2012). Nectin4 Is an Epithelial Cell Receptor for Canine Distemper Virus and Involved in Neurovirulence. J. Virol..

[11] Noyce R.S., Delpeut S., Richardson C.D. (2013). Dog nectin-4 is an epithelial cell receptor for canine distemper virus that facilitates virus entry and syncytia formation. Virology.

[12] Von Messling V., Milosevic D., Cattaneo R. (2004). Tropism illuminated: Lymphocyte-based pathways blazed by lethal morbillivirus through the host immune system. Proc. Natl. Acad. Sci. USA.

[13] Santiago C., Björling E., Stehle T., Casasnovas J.M. (2002). Distinct kinetics for binding of the CD46 and SLAM receptors to overlapping sites in the measles virus hemagglutinin protein. J. Biol. Chem..

[14] Hashiguchi T., Kajikawa M., Maita N., Takeda M., Kuroki K., Sasaki K., Kohda D., Yanagi Y., Maenaka K. (2007). Crystal structure of measles virus hemagglutinin provides insight into effective vaccines. Proc. Natl. Acad. Sci. USA.

[15] Khosravi M., Bringolf F., Röthlisberger S., Bieringer M., Schneider-Schaulies J., Zurbriggen A., Origgi F., Plattet P. (2016). Canine Distemper Virus Fusion Activation: Critical Role of Residue E123 of CD150/SLAM. J. Virol..

[16] Niwa H., Yamamura K., Miyazaki J. (1991). Efficient selection for high-expression transfectants with a novel eukaryotic vector. Gene.

[17] Hashiguchi T., Ose T., Kubota M., Maita N., Kamishikiryo J., Maenaka K., Yanagi Y. (2011). Structure of the measles virus hemagglutinin bound to its cellular receptor SLAM. Nat. Struct. Mol. Biol..

[18] Ono N., Tatsuo H., Hidaka Y., Aoki T., Minagawa H., Yanagi Y. (2001). Measles Viruses on Throat Swabs from Measles Patients Use Signaling Lymphocytic Activation Molecule (CDw150) but Not CD46 as a Cellular Receptor. J. Virol..

[19] Seki F., Ono N., Yamaguchi R., Yanagi Y. (2003). Efficient Isolation of Wild Strains of Canine Distemper Virus in Vero Cells Expressing Canine SLAM (CD150) and Their Adaptability to Marmoset B95a Cells. J. Virol..

[20] Otsuki N., Sekizuka T., Seki F., Sakai K., Kubota T., Nakatsu Y., Chen S., Fukuhara H., Maenaka K., Yamaguchi R. (2013). Canine distemper virus with the intact C protein has the potential to replicate in human epithelial cells by using human nectin4 as a receptor. Virology.

[21] Lan N.T., Yamaguchi R., Inomata A., Furuya Y., Uchida K., Sugano S., Tateyama S. (2006). Comparative analyses of canine distemper viral isolates from clinical cases of canine distemper in vaccinated dogs. Vet Microbiol..

[22] Yamaguchi R., Iwai H., Ueda K. (1988). Variation of Virulence and Other Properties among Sendai Virus Strains. Microbiol. Immunol..

[23] Riazantseva N.E. (1956). Experimental measles in puppies. Zh Mikrobiol. Immunobiol..

[24] Ohno S., Seki F., Ono N., Yanagi Y. (2003). Histidine at position 61 and its adjacent amino acid residues are critical for the ability of SLAM (CD150) to act as a cellular receptor for measles virus. J. Gen. Virol..

[25] Vongpunsawad S., Oezgun N., Braun W., Cattaneo R. (2004). Selectively receptor-blind measles viruses: Identification of residues necessary for SLAM- or CD46-induced fusion and their localization on a new hemagglutinin structural model. J. Virol..

[26] Von Messling V., Oezguen N., Zheng Q., Vongpunsawad S., Braun W., Cattaneo R. (2005). Nearby Clusters of Hemagglutinin Residues Sustain SLAM-Dependent Canine Distemper Virus Entry in Peripheral Blood Mononuclear Cells. J. Virol..

[27] Zipperle L., Langedijk J.P., Orvell C., Vandevelde M., Zurbriggen A., Plattet P. (2010). Identification of key residues in virulent canine distemper virus hemagglutinin that control CD150/SLAM-binding activity. J. Virol..

[28] An D.J., Yoon S.H., Park J.Y., No I.S., Park B.K. (2008). Phylogenetic characterization of canine distemper virus isolates from naturally infected dogs and a marten in Korea. Vet. Microbiol..

[29] Sun Z., Li A., Ye H., Shi Y., Hu Z., Zeng L. (2010). Natural infection with canine distemper virus in hand-feeding Rhesus monkeys in China. Vet. Microbiol..

[30] Qiu W., Zheng Y., Zhang S., Fan Q., Liu H., Zhang F., Wang W., Liao G., Hu R. (2011). Canine distemper outbreak in rhesus monkeys, China. Emerg. Infect. Dis..

[31] Sakai K., Nagata N., Ami Y., Seki F., Suzaki Y., Iwata-Yoshikawa N., Suzuki T., Fukushi S., Mizutani T., Yoshikawa T. (2013). Lethal canine distemper virus outbreak in cynomolgus monkeys in Japan in 2008. J. Virol..

[32] Bieringer M., Han J.W., Kendl S., Khosravi M., Plattet P., Schneider-Schaulies J. (2013). Experimental Adaptation of Wild-Type Canine Distemper Virus (CDV) to the Human Entry Receptor CD150. PLoS ONE.

