# Supplementary material for: Specificity of Morbillivirus Hemagglutinins to Recognize SLAM of Different Species
Source: Viruses. 2019 Aug 19;11(8):761. doi: 10.3390/v11080761 (PMC6722581; doi:10.3390/v11080761)
Supplement: Supplementary file 1 [file viruses-11-00761-s001.pdf]

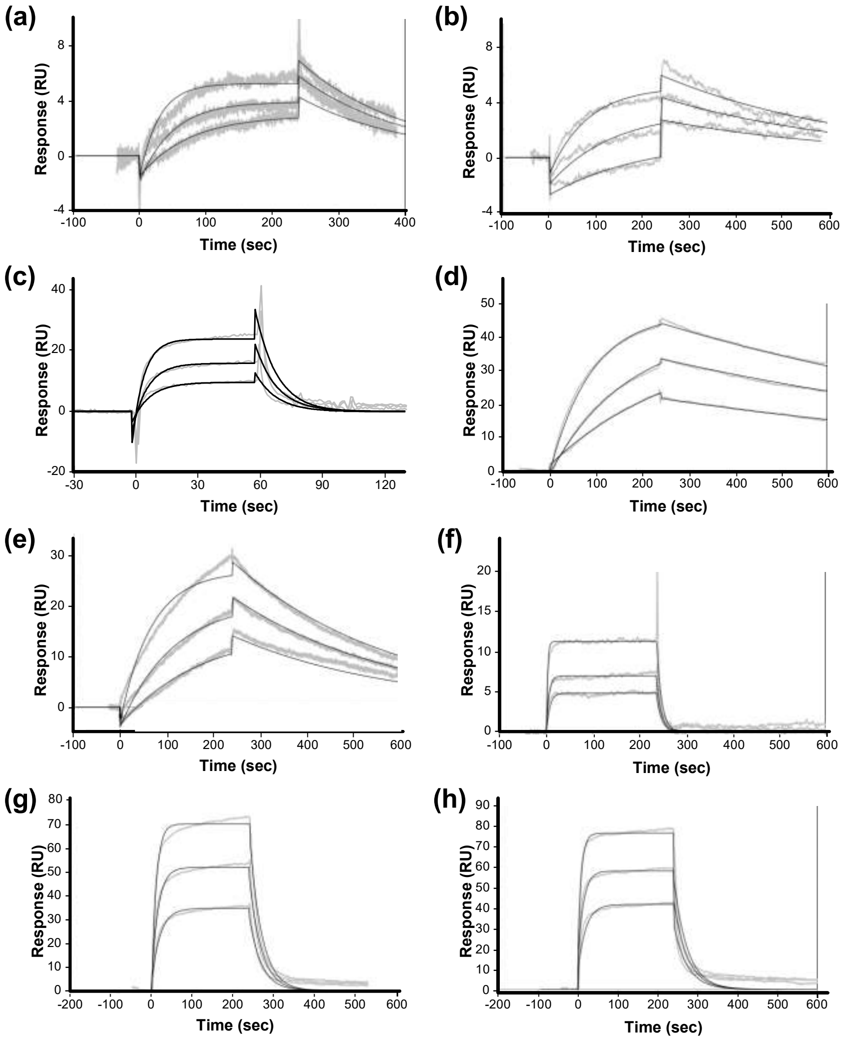

Figure S1. Kinetic Analysis of SLAM proteins against MV-H and CDV-H from the wild-type and vaccine strain. (**a**) MV-Hwt vs human SLAM. (**b**) MV-Hwt vs tamarin SLAM. (**c**) MV-Hwt vs dog SLAM. (**d**) MV-Hvac vs tamarin SLAM. (**e**) MV-Hvac vs human SLAM. (**f**) MV-Hvac vs dog SLAM. (**g**) CDV-Hwt vs dog SLAM. (**h**) CDV-Hvac vs dog SLAM.
